# Diagnostic Accuracy and Concordance of Hysteroscopy for Detecting Premalignant and Malignant Endometrial Lesions: A Retrospective Study From a Tertiary Center in Mexico

**DOI:** 10.7759/cureus.108517

**Published:** 2026-05-08

**Authors:** Fernando J Interian-Alvarez, Brayan J Ortiz-Villanueva, Luz M Bravo-Rodriguez, Alejandra G Lopez-Chavez, Hector A Hurtado Bravo

**Affiliations:** 1 Faculty of Medicine, Universidad Autónoma de Yucatán, Mérida, MEX; 2 Faculty of Medicine, La Salle University, Mexico City, MEX; 3 Department of Gynecological Endoscopic Surgery, Hospital Regional Adolfo López Mateos, Institute for Social Security and Services for State Workers (ISSSTE), Mexico City, MEX

**Keywords:** diagnostic accuracy, endometrial hyperplasia, endometrial neoplasms, hysteroscopy, uterine hemorrhage

## Abstract

Background

Hysteroscopy is widely regarded as the gold standard for the evaluation of intrauterine pathology due to its ability to provide direct visualization of the endometrium. In patients with suspected endometrial disease, the primary clinical objective is to exclude malignancy and identify premalignant precursor lesions such as endometrial intraepithelial neoplasia (EIN). However, the diagnostic performance of visual hysteroscopic impression compared to histopathology remains variable. This study aimed to assess the diagnostic accuracy and concordance between hysteroscopic findings and histopathological results in a tertiary care center in Mexico.

Methods

An observational, cross-sectional, retrospective analytical study was conducted, including 397 patients who underwent hysteroscopy between January 2024 and December 2025. The surgeon’s visual impression (index test) was compared with the histopathological diagnosis obtained from directed biopsy (reference standard). Diagnostic performance was evaluated through sensitivity, specificity, positive predictive value (PPV), and negative predictive value (NPV). Concordance between both methods was assessed using Cohen’s kappa coefficient.

Results

The mean age of the study population was 48.8 years, and 36.3% were postmenopausal. Hysteroscopic findings suggested malignancy in 9.8% of cases. A total of 41 patients were identified with high-risk endometrial pathology, including endometrial hyperplasia and EIN/atypical endometrial hyperplasia (AEH). Hysteroscopy demonstrated a sensitivity of 60.97% and a specificity of 96.06%. The PPV was 64.1%, while the NPV reached 95.53%. The Cohen’s kappa coefficient was 0.541, indicating moderate agreement between hysteroscopic impression and histopathological findings.

Conclusions

Hysteroscopy is a valuable diagnostic tool for excluding high-risk endometrial pathology, given its high specificity and NPV. However, its moderate sensitivity limits its reliability for definitive diagnosis of premalignant and malignant lesions. These findings highlight the necessity of performing directed biopsies for histopathological confirmation, particularly in cases with suspicious visual findings.

## Introduction

Hysteroscopy has emerged as the gold standard for the evaluation of intrauterine pathology, allowing direct visualization of the endometrial cavity and facilitating targeted biopsy of suspicious lesions. Its evolution from an invasive operative procedure to a minimally invasive diagnostic tool has significantly improved the accuracy and efficiency of gynecological assessment, particularly in patients presenting with abnormal uterine bleeding (AUB), one of the most common indications for gynecologic consultation worldwide [1-3].

The clinical priority in patients with suspected endometrial pathology is the timely exclusion of malignancy and the identification of premalignant lesions, particularly endometrial intraepithelial neoplasia (EIN) or atypical endometrial hyperplasia (AEH). These entities represent a critical biological threshold due to their well-established progression to endometrial carcinoma, with an estimated annual progression risk of approximately 8%. Furthermore, a substantial proportion of patients diagnosed preoperatively with atypical hyperplasia are found to have concurrent endometrial carcinoma in definitive surgical specimens [4-6].

Although transvaginal ultrasound is widely used as a first-line diagnostic tool, its limited specificity often necessitates further evaluation. Similarly, blind sampling techniques, such as dilatation and curettage, may fail to detect focal lesions in a significant proportion of cases. In contrast, hysteroscopy enables direct inspection of the endometrial surface and allows for targeted biopsies, thereby improving diagnostic yield and reducing the likelihood of missed pathology [3,6].

Hysteroscopic evaluation also provides characteristic visual patterns that may suggest malignant transformation. These include atypical vascularization with irregular branching, papillary or nodular projections, friable tissue, and areas of focal necrosis or hemorrhage [7,8]. Despite these recognizable features, the reliability of hysteroscopic visual impression alone remains controversial, particularly in distinguishing benign from premalignant or malignant lesions.

Previous studies have reported high specificity but variable sensitivity for hysteroscopic diagnosis of endometrial pathology, suggesting that while it may be effective in ruling out disease, its capacity to confirm malignancy based solely on visual findings is limited [3]. This diagnostic limitation has important clinical implications, as misclassification may lead to delayed diagnosis or inappropriate management.

In addition, the epidemiological profile of endometrial pathology in the Mexican population, characterized by a high prevalence of metabolic risk factors such as obesity, diabetes mellitus, and hypertension, underscores the need for accurate diagnostic strategies in specialized healthcare settings. Regional data have reported a significant burden of endometrial pathology, with polyps, endometritis, and adenocarcinoma among the most frequent findings [9,10].

Given these considerations, the present study aimed to evaluate the diagnostic accuracy and concordance between hysteroscopic visual findings and histopathological results for the detection of premalignant and malignant endometrial lesions in a tertiary care center in Mexico City.

## Materials and methods

Study design and setting

An observational, cross-sectional, retrospective analytical study was conducted at a tertiary care center in Mexico City, Mexico. The study included patients who underwent diagnostic or operative hysteroscopy between January 2024 and December 2025.

Study population

A non-probabilistic consecutive sampling method was used. A total of 526 digital medical records were initially reviewed. After applying the selection criteria, 397 patients with complete clinical, hysteroscopic, and histopathological data were included in the final analysis.

Eligibility criteria

Inclusion Criteria

Patients post-menarche with a clinical indication for hysteroscopy, performed at the study center during the defined period, and with complete records including hysteroscopic findings and corresponding histopathological reports from directed biopsy.

Exclusion Criteria

Cases with histopathological reports classified as “insufficient tissue” or “non-diagnostic.”

Elimination Criteria

**Figure 1:**
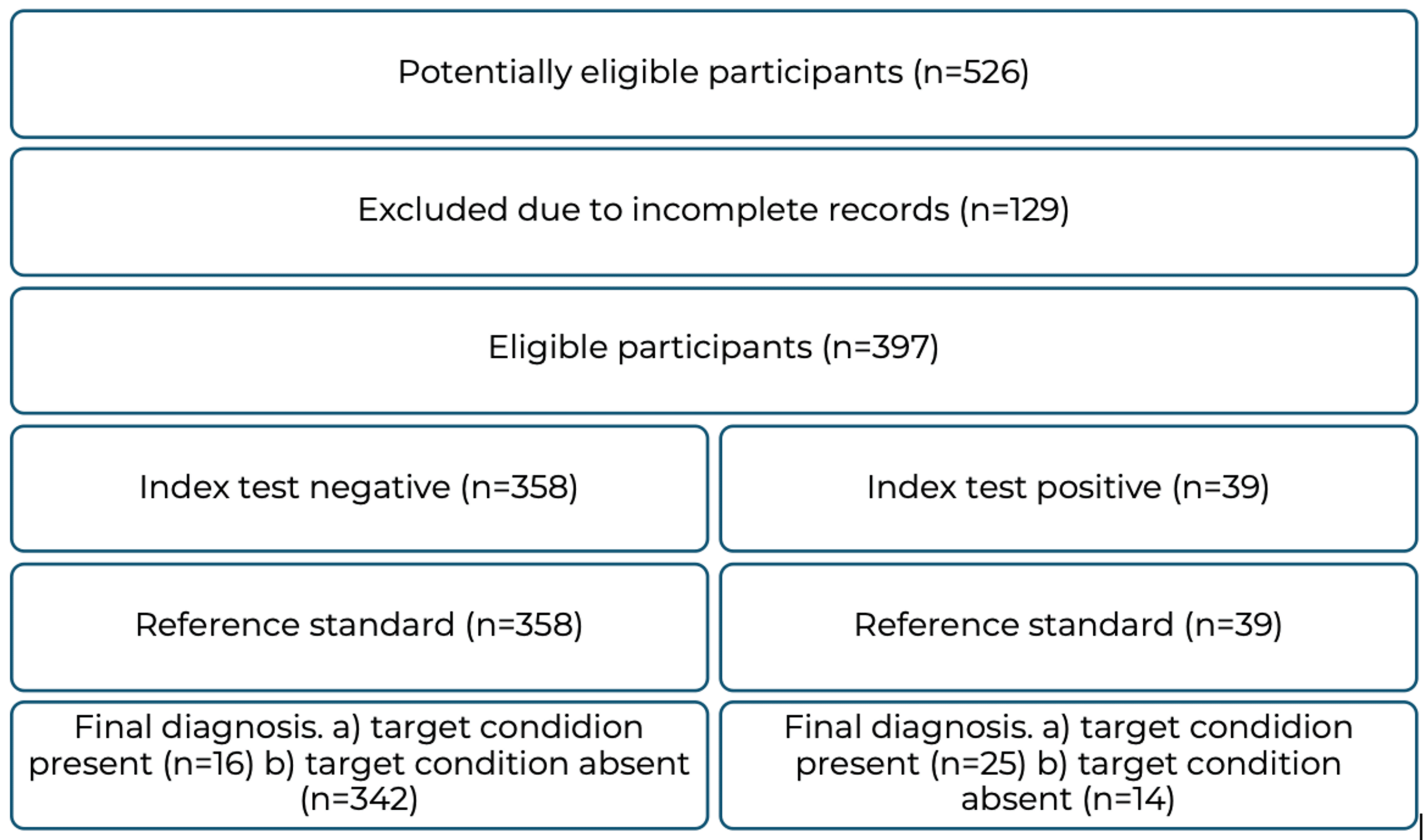
Study flow diagram illustrating patient selection and diagnostic classification pathway Flow diagram summarizing the selection process of the study population and the classification of cases according to hysteroscopic findings and histopathological outcomes. The diagram illustrates the sequential allocation of patients into index test categories and their corresponding reference standard results, allowing visualization of the distribution of true positives, false positives, true negatives, and false negatives used for diagnostic accuracy analysis.

Medical records with incomplete data for key variables, including age, menopausal status, or comorbidities (Figure 1).

Data collection

Clinical records and surgical reports were systematically reviewed. The following variables were collected:

- Sociodemographic and clinical variables: age, menopausal status, and comorbidities (diabetes mellitus and systemic arterial hypertension).

- Index test (hysteroscopy): The hysteroscopic visual impression was recorded in all cases based on the findings documented in the clinical and surgical reports. All patients underwent hysteroscopy as part of their diagnostic evaluation. The endometrial appearance was classified as either benign-looking or suspicious/malignant-looking.

The classification was based on previously described hysteroscopic criteria, including features such as irregular or diffuse endometrial thickening, polypoid or papillary structures, abnormal vascular patterns, glandular cystic changes, and architectural disorganization suggestive of endometrial hyperplasia or EIN/AEH.

Hysteroscopic procedures were performed by experienced gynecological surgeons at the study center. Although multiple operators were involved, variability was mitigated by the use of standardized institutional reporting formats and consistent categorization criteria documented in the clinical records. Furthermore, all procedures were conducted in a tertiary care setting by trained personnel with comparable levels of expertise, which reduces interobserver variability.

- Reference standard (histopathology): definitive diagnosis established by microscopic evaluation of directed biopsy specimens.

Hysteroscopic procedure

All hysteroscopic procedures were performed using a 4-mm hysteroscope with a 30° fore-oblique lens. Normal saline solution was used as the distension medium in all cases. Procedures were conducted in both office-based and operating room settings according to clinical indication. In postmenopausal patients, cervical preparation with prostaglandin E1 (misoprostol) was administered prior to the procedure to facilitate cervical dilation.

Statistical analysis

Diagnostic performance of hysteroscopy was evaluated by comparing the surgeon’s visual impression (index test) with histopathological findings (reference standard). A 2 × 2 contingency table was constructed, and the following indicators were calculated: sensitivity, specificity, positive predictive value (PPV), and negative predictive value (NPV).

Concordance between hysteroscopic findings and histopathology was assessed using Cohen’s kappa coefficient. The strength of agreement was interpreted as follows: values <0.20 indicated poor agreement, 0.21-0.40 fair agreement, 0.41-0.60 moderate agreement, 0.61-0.80 substantial agreement, and >0.80 almost perfect agreement.

Confidence intervals (95% CI) were calculated when applicable.

Sample size calculation

The required sample size was estimated using the formula for proportions in an infinite population, assuming a prevalence of endometrial pathology of 37%, a 5% margin of error, and a 95% confidence level. The minimum required sample size was calculated as 359 patients. The final sample of 397 patients exceeded this requirement.

Ethical considerations

This study was conducted in accordance with the Declaration of Helsinki. Given its retrospective design, the requirement for informed consent was waived by the Institutional Ethics and Research Committee. Patient confidentiality and data anonymization were strictly maintained.

## Results

A total of 526 digital medical records from a tertiary care center in Mexico City were initially reviewed. After applying the inclusion and exclusion criteria, 397 patients with complete clinical, hysteroscopic, and histopathological data were included in the final analysis.

The study population ranged in age from 25 to 81 years, with a mean age of 48.8 years. Of these, 144 patients (36.3%) were postmenopausal. Regarding comorbidities, 54 patients (13.6%) had diabetes mellitus, and 76 patients (19.1%) had systemic arterial hypertension.

The clinical indication for hysteroscopy differed according to menopausal status. All postmenopausal patients (n = 144) underwent evaluation due to postmenopausal bleeding and/or abnormal endometrial findings on transvaginal ultrasound, including endometrial thickening or suspected intrauterine lesions. Among premenopausal patients (n = 253), 92% were referred for AUB, while the remaining 8% underwent hysteroscopy for other indications, including suspected structural abnormalities based on imaging studies.

Hysteroscopic findings were classified as benign-looking in 358 cases (90.1%) and suspicious/malignant-looking in 39 cases (9.8%), based on the descriptions recorded in the clinical reports. Due to the retrospective nature of the study, detailed morphological characteristics were not consistently documented across all records, and therefore, a more granular classification of hysteroscopic features suggestive of malignancy was not feasible.

Histopathological analysis identified 41 patients with high-risk endometrial pathology, including endometrial hyperplasia and EIN/AEH, corresponding to a prevalence of 10.3% in the study population. Among these cases, 10 were diagnosed as endometrial carcinoma, while the remaining corresponded to premalignant lesions.

When correlating hysteroscopic findings with histopathological results, 25 of the 39 patients (64.1%) with suspicious or malignant-looking hysteroscopy were confirmed to have high-risk endometrial pathology. Conversely, 16 patients with benign-looking hysteroscopy were found to have positive histopathological findings, highlighting the presence of false-negative cases associated with visual assessment.

Diagnostic accuracy

**Figure 2:**
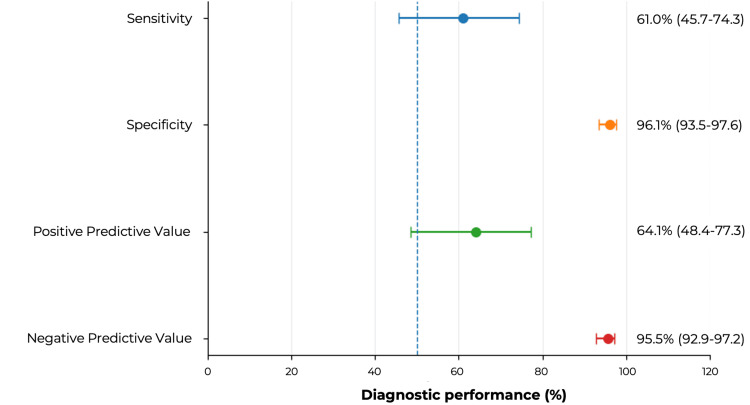
Diagnostic performance of hysteroscopy for premalignant and malignant endometrial lesions Graphical representation of the diagnostic performance of hysteroscopy for detecting premalignant and malignant endometrial lesions. Point estimates are displayed alongside their corresponding 95% confidence intervals, illustrating the variability and precision of each metric. The plot highlights the asymmetry between sensitivity and specificity, as well as the narrow dispersion observed in specificity and negative predictive value compared to the wider intervals of sensitivity and positive predictive value, reflecting differences in diagnostic reliability across parameters.

The diagnostic performance of hysteroscopy is illustrated in Figure 2. Hysteroscopy demonstrated a sensitivity of 60.97% and a specificity of 96.06%. The PPV was 64.1%, while the NPV reached 95.53%.

Concordance analysis showed a Cohen’s kappa coefficient of 0.541, indicating a moderate level of agreement between hysteroscopic impression and histopathological diagnosis.

Contingency table

The distribution of hysteroscopic findings in relation to histopathological results is presented in Table 1.

**Table 1:** Contingency table of hysteroscopic findings versus histopathological diagnosis Cross-tabulation of hysteroscopic visual classification and corresponding histopathological outcomes used to derive diagnostic accuracy measures. The table displays the distribution of true positive, false positive, true negative, and false negative cases, providing the basis for the calculation of sensitivity, specificity, and predictive values. This matrix also illustrates the relative proportion of misclassified cases, particularly false negatives, which contribute to the observed limitations in sensitivity.

	Histopathology Positive	Histopathology Negative	Total
Hysteroscopy Positive	25	14	39
Hysteroscopy Negative	16	342	358
Total	41	356	397

## Discussion

The present study evaluated the diagnostic performance and concordance of hysteroscopic visual impression compared with histopathological findings for the detection of high-risk endometrial pathology in a tertiary care setting. Our results demonstrated a sensitivity of 60.97% and a specificity of 96.06%, with a PPV of 64.1% and a notably high NPV of 95.53%. Additionally, concordance analysis yielded a Cohen’s kappa coefficient of 0.541, indicating a moderate level of agreement between hysteroscopic assessment and the reference standard.

These findings reinforce the concept that hysteroscopy is highly reliable for ruling out significant endometrial pathology, as reflected by its high specificity and NPV. In clinical terms, a benign-appearing endometrium under hysteroscopic visualization is strongly associated with the absence of high-risk lesions. This observation is consistent with previous studies reporting high specificity and strong negative predictive performance of hysteroscopy in excluding malignancy, particularly in patients with AUB [11,12].

Similarly, Gan et al. demonstrated that hysteroscopy has a high concordance rate with histopathology (up to 88.8%), with particularly strong performance in ruling out malignancy, despite moderate sensitivity for hyperplasia, findings that closely mirror those observed in our cohort [12].

However, the sensitivity observed in our study was moderate, indicating that a proportion of patients with premalignant or malignant pathology may not be identified through visual assessment alone. This limitation has been consistently reported in the literature, particularly for endometrial hyperplasia and early malignant changes, where hysteroscopic interpretation may be challenging and subject to misclassification [3,11]. In line with this, De Franciscis et al. reported that although hysteroscopy demonstrates high sensitivity in experienced hands, its PPV remains limited due to the subjective nature of morphological interpretation and overlap between benign and premalignant patterns [13].

The moderate concordance observed in our study (kappa = 0.541) further highlights the inherent variability of hysteroscopic interpretation. This variability is largely attributable to the subjective evaluation of visual patterns and operator experience. Dueholm et al. demonstrated that even structured hysteroscopic scoring systems, although improving diagnostic accuracy, still show only moderate interobserver agreement (kappa = ~0.56), supporting the findings of our study [14].

Importantly, structured evaluation systems have been proposed to improve diagnostic performance. Dueholm et al. showed that standardized assessment of surface characteristics, vascular patterns, and necrotic features significantly improves the prediction of malignancy, achieving higher diagnostic accuracy than subjective evaluation alone [14]. This suggests that implementing structured hysteroscopic criteria could potentially enhance reproducibility and diagnostic precision in clinical practice.

From a broader perspective, evidence from systematic reviews further supports the diagnostic utility of hysteroscopy. Riemma et al., in a diagnostic test accuracy meta-analysis, reported pooled sensitivity and specificity values of approximately 84% and 89%, respectively, with an area under the curve (AUC) of 0.93, indicating high overall diagnostic accuracy for hysteroscopic criteria when standardized definitions are applied [15]. However, the authors emphasize that histopathological confirmation remains necessary, particularly in cases with suspicious or ambiguous findings, due to the risk of false positives and variability in visual interpretation.

Additionally, evidence from studies evaluating inflammatory endometrial conditions highlights the complementary role of hysteroscopy and biopsy. Ikramova et al. reported that while hysteroscopy provides high sensitivity for detecting endometrial abnormalities, its diagnostic accuracy is significantly enhanced when combined with targeted biopsy, reinforcing the concept that hysteroscopy should not be used as a standalone diagnostic modality [16].

From a clinical perspective, our findings strongly support a combined diagnostic approach. While hysteroscopy offers clear advantages in real-time visualization and targeted biopsy guidance, reliance solely on visual impression may lead to missed diagnoses, particularly in early or focal lesions. Therefore, histopathological confirmation remains essential, especially in patients with risk factors or persistent symptoms, as also emphasized in current clinical recommendations [6,9].

The epidemiological context of our population is also relevant. The prevalence of high-risk endometrial pathology in our cohort (10.3%) reflects a clinically meaningful burden of disease in a tertiary care setting. Additionally, the presence of metabolic comorbidities, such as diabetes mellitus and hypertension, is consistent with established risk factors for endometrial pathology and may influence both disease prevalence and diagnostic performance [10]. Furthermore, as previously reported, even premenopausal women with AUB may harbor premalignant lesions, particularly in the presence of associated risk factors, underscoring the need for accurate and systematic diagnostic strategies [17].

This study has several limitations. First, its retrospective design may introduce selection and information bias. Second, the absence of blinding between hysteroscopic findings and histopathological evaluation could have influenced interpretation. Third, the study was conducted in a tertiary care center, which may limit generalizability to lower-risk or screening populations.

Despite these limitations, this study provides valuable real-world evidence and contributes to the existing literature by confirming that hysteroscopy is a highly specific diagnostic tool with excellent NPV, but limited sensitivity. The integration of structured hysteroscopic evaluation with histopathological confirmation remains essential to optimize diagnostic accuracy and ensure appropriate clinical decision-making.

## Conclusions

Hysteroscopic visual assessment demonstrated high specificity (96.06%) and an excellent NPV (95.53%), supporting its role as a reliable tool for ruling out high-risk endometrial pathology in clinical practice. However, the observed sensitivity of 60.97% indicates a limited capacity to detect all cases of endometrial hyperplasia and EIN/AEH based solely on visual impression, highlighting a non-negligible proportion of false-negative results. The moderate concordance observed between hysteroscopic findings and histopathological diagnosis (Cohen’s kappa = 0.541) further underscores the variability in visual interpretation and reinforces the need for objective confirmation. Taken together, these findings indicate that while hysteroscopy provides strong diagnostic reassurance when findings are benign, it should not be considered sufficient as a standalone diagnostic modality.

Therefore, directed endometrial biopsy remains essential for definitive diagnosis, particularly in patients with suspicious hysteroscopic features or persistent clinical concern. This combined approach optimizes diagnostic accuracy and minimizes the risk of underdiagnosis in premalignant and high-risk endometrial conditions.
